# Symptomatic Paratracheal Air Cyst Mimicking Esophageal Diverticulum: A Rare Presentation and a Diagnostic Challenge

**DOI:** 10.7759/cureus.96278

**Published:** 2025-11-07

**Authors:** Ghala Almezrem, Mishal M AlMutairi

**Affiliations:** 1 Department of Otorhinolaryngology–Head and Neck Surgery, Farwaniya Hospital, Farwaniya, KWT

**Keywords:** computed tomography (ct ), esophageal diverticulum, gastrograffin swallow test, paratracheal air cyst, tracheal diverticula

## Abstract

A 72-year-old woman presented with symptomatic acute odynophagia, right-sided neck pain, and fever. Initial imaging with computed tomography (CT) revealed the right para-esophageal collection suggestive of a complicated lateral esophageal diverticulum. A gastrografin swallow study showed an external indentation on the right lateral aspect of the upper third of the esophagus, with subsequent relative luminal narrowing, yet no evidence of wall infiltration or esophageal diverticulum was noted, reaching the diagnosis of para-tracheal air cyst with a superadded inflammatory process rather than esophageal diverticulum. The patient underwent bronchoscopy and upper endoscopy evaluations later, which were both reported as normal studies, with no detectable opening into either the airway or the esophagus. The patient was managed conservatively with intravenous antibiotics and achieved complete resolution of symptoms. This case highlights a rare symptomatic presentation of paratracheal air cyst (PTAC) mimicking an esophageal diverticulum and emphasizes the importance of multimodal imaging and endoscopic evaluation for accurate diagnosis. Awareness of this entity is essential to avoid misdiagnosis and unnecessary surgical intervention.

## Introduction

Paratracheal air cysts (PTACs) are rare, air-filled lesions located on the right posterior side of the tracheal wall and are often discovered incidentally on imaging. Although typically asymptomatic, they may occasionally present with compressive or inflammatory symptoms, leading to diagnostic confusion [1]. Studies showed that pathologically, these lesions are unclear, and they can be congenital or acquired. Paratracheal air cysts may be a common CT finding that occurs in a predictable location [2].

The pathogenesis of PTACs remains incompletely understood. Some are thought to represent small tracheal diverticula, lined by ciliated respiratory epithelium, while others may arise from lymphoepithelial or bronchogenic origin. Whether congenital or acquired, their morphology and communication with the tracheal lumen can vary, compounding the challenge of classification [3]. In a study using multidetector CT, PTACs have been identified in up to about 8% of subjects [4]. In fact, Buterbaugh and Erly pointed out that right paratracheal air cysts are a “common CT finding” that appear in a predictable posterolateral location, though without necessarily clinical relevance [5]. 

Although most PTACs remain asymptomatic, rare presentations with cough, neck discomfort, hoarseness, dysphagia, or odynophagia have been documented [6]. These symptoms often provoke an extensive differential diagnosis, including esophageal diverticula, Zenker’s diverticulum, laryngocele, or mediastinal cysts, especially when imaging is equivocal [6].

In this report, we describe the clinical journey of a 72-year-old woman who presented with odynophagia and right-sided neck pain. Her case underwent a demanding diagnostic workup that initially suggested an esophageal diverticulum before advanced imaging ultimately confirmed a PTAC without demonstrable communication to the trachea or esophagus. This unusual symptomatic presentation underscores the necessity of a systematic, multidisciplinary diagnostic strategy and judicious interpretation of radiographic findings to arrive at the correct diagnosis and optimize patient management.

## Case presentation

**Figure 1:**
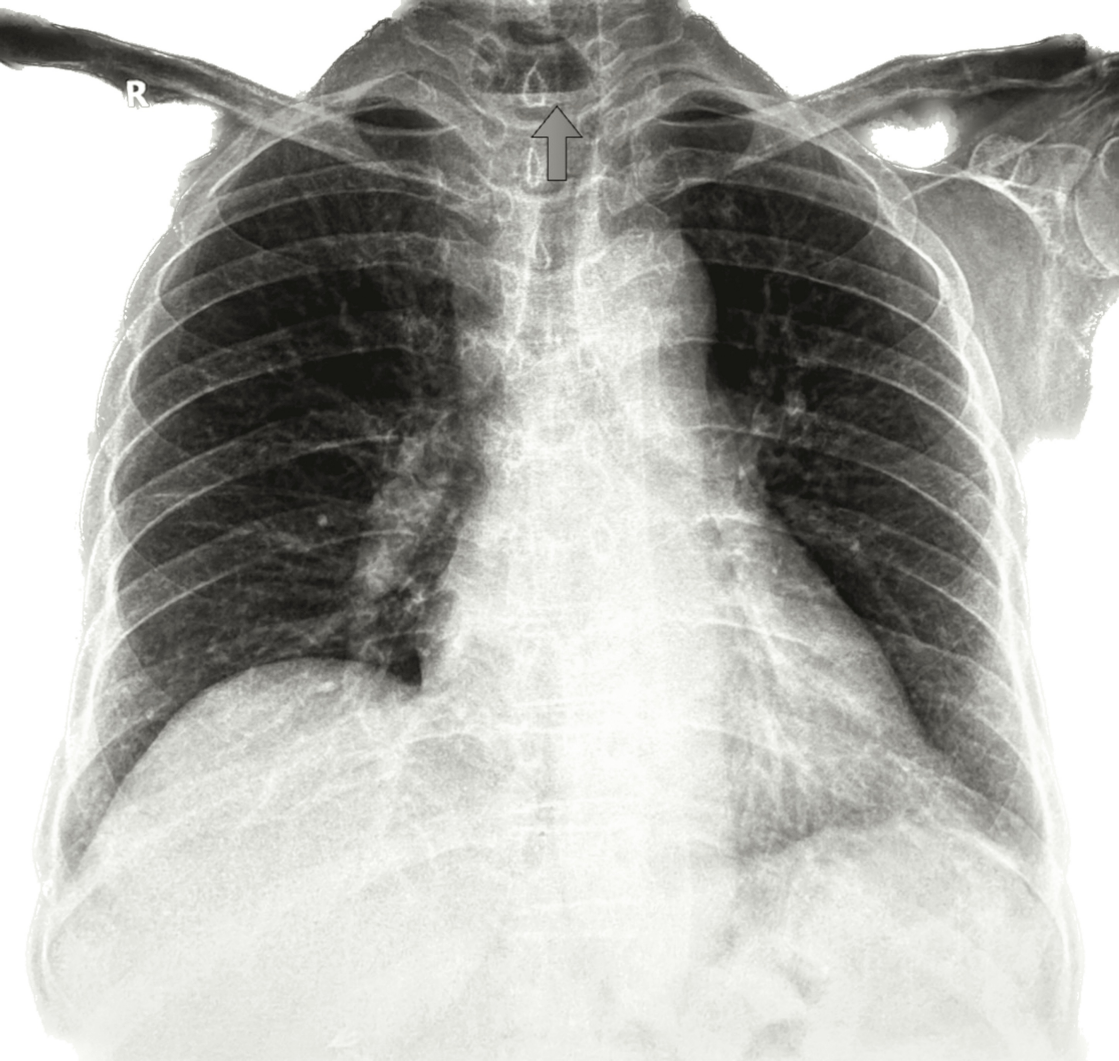
X-ray of the chest and cervical spine Showed the prevertebral post-tracheal space is widened in this current study, measuring 3.1 cm, with a lucent area that is seen in previous X-rays tracing back to 2008 with no air-fluid level present.

A 72-year-old female presented to Farwaniya Hospital in Kuwait with a history of fever and intermittent non-productive cough for a week. Her symptoms were complicated by the sudden onset of severe odynophagia (painful swallowing) associated with right-sided neck pain for three days. The patient denied any previous episodes of similar symptoms, recent trauma, dyspnea, or hoarseness of voice. She reported a history of self-induced vomiting on several occasions due to chronic heartburn. Her past medical history included hypothyroidism, for which she was on levothyroxine 50 micrograms daily, type 2 diabetes mellitus, and hypertension. She was allergic to penicillin, a non-smoker, and did not consume alcohol. Family history was unremarkable. On examination, moderate tenderness was appreciated upon palpation of the right lateral aspect of the neck. Deep palpation over the right medial region above the clavicle revealed a firm, minimally sized swelling with transmitted pulsation. No cervical lymphadenopathy was appreciated. Fiberoptic nasal laryngoscopy revealed a normal larynx with bilaterally mobile vocal cords. Chest and cervical spine X-rays were obtained (Figure 1). The chest X-ray was unremarkable, while the cervical spine image demonstrated widening of the prevertebral post-tracheal space measuring approximately 3.1 cm, consistent with findings seen in previous imaging from 2008.

Initial laboratory investigations revealed leukocytosis with a white blood cell count (WBC) of 17.9 × 10⁹/L. Inflammatory markers were elevated, with an erythrocyte sedimentation rate (ESR) of 29 mm/hr, a C-reactive protein (CRP) of 230 mg/L, and a procalcitonin (PCT) of 0.205 ng/mL. The patient was started on intravenous clindamycin (600 mg every eight hours). A summary of the laboratory investigations is shown in Table 1.

**Table 1:** Summary of laboratory investigations.

Test Name	Result Value	Reference Range	Unit	Interpretation
White Blood Cell Count (WBC)	17.9	3.9 - 11.1	×10⁹/L	High
Erythrocyte Sedimentation Rate (ESR)	29	0 - 20	mm/hr	High
C-Reactive Protein (CRP)	230	0-8	mg/L	High
Procalcitonin (PCT)	0.205	0.02-0.046	ng/mL	High

**Figure 2:**
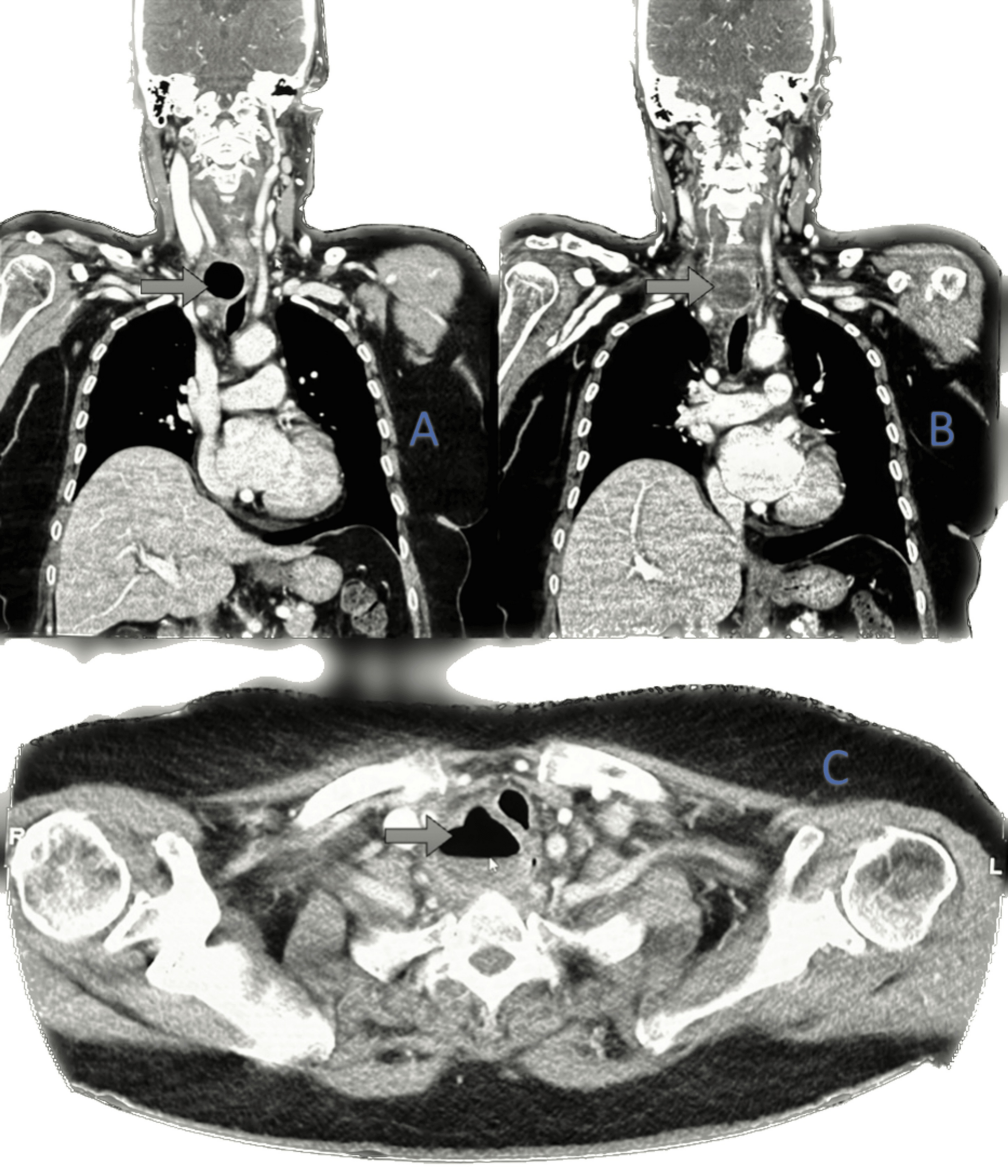
Contrast-enhanced CT scan Showed the right para-esophageal region at the thoracic inlet opposite to the C7-D1 vertebral levels, a well-defined, thick-walled, marginally enhanced collection of air-fluid level. (a) coronal section showing air-filled lesion (b) coronal section showing fluid-filled lesion (c) axial section showing Medially compressing and displacing the esophagus to the contralateral side, also seen as mid-displacing the trachea to the left anterolateral aspect.

A computed tomography (CT) scan of the neck and chest with contrast revealed a well-defined, thick-walled, marginally enhancing lesion with an air-fluid level located at the right para-esophageal region at the thoracic inlet opposite to the C7-D1 vertebral levels, measuring 4 × 3.7 × 4.1 cm (anteroposterior × transverse × height). Mild mural thickening and edema were noted in the related segment of the esophagus, with mediastinal fat stranding. The lesion compressed and displaced the esophagus contralaterally and caused mild displacement of the trachea to the left anterolateral aspect, as shown in Figures 2a-2c.

**Figure 3:**
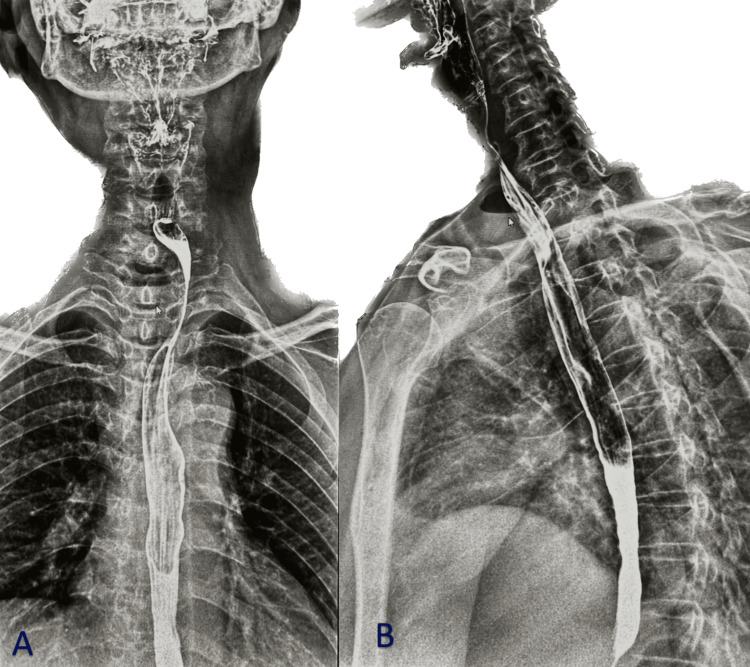
Gastrograffin swallow meal Both (A and B) showing air-fluid leveling, causing external indentation on the right lateral aspect of the upper third of the esophagus with subsequent relative luminal narrowing

A review of the patient’s prior chest X-rays dating back to 2008 revealed a right paratracheal air pocket at the same anatomical level, suggesting a chronic diverticular or cystic lesion rather than an acute abscess. Based on these findings, further evaluation was recommended. A Gastrografin (barium) swallow study was subsequently performed (Figure 3), showing a soft tissue shadow with an air-fluid level causing external indentation on the right lateral wall of the upper third of the esophagus with mild luminal narrowing. No evidence of mucosal defect or communication with the esophageal lumen was seen, suggesting a paratracheal air cyst with superimposed inflammation.

Bronchoscopic and upper endoscopic examinations were both unremarkable, revealing no visible communication with either the trachea or esophagus. The patient’s symptoms resolved completely following the antibiotic course. She was discharged in stable condition with instructions to obtain a follow-up magnetic resonance imaging (MRI) of the neck and chest with oral contrast to evaluate the cystic lesion further and to assess the need for elective surgical excision to prevent future complications.

## Discussion

PTAC refers to an air-filled lesion arising from the right posterior aspect of the trachea with uncertain etiology. These lesions are most often detected as incidental findings on routine chest computed tomography (CT) scans, and their true prevalence may be underestimated as most patients remain asymptomatic. Previous studies reported that tracheal compression or secondary infection of the cyst may occasionally occur [7].

In a series reported by Goo et al., involving 65 patients with PTACs, 98% of the lesions were in the right paratracheal region. A definitive communication between the cyst and the tracheal lumen was identified in only five patients (8%) [2]. The incidence of PTACs has been reported as 3.7%, which is higher than earlier estimates. In low-dose screening chest CT, 98.7% of the identified PTACs were right-sided, with only a single lesion found on the left side [8].

The strong right-sided predominance may be explained by the supportive effect of the esophagus and the aortic arch on the left side of the trachea. This anatomical support likely prevents PTACs formation on the left, whereas the relatively unsupported right side is more vulnerable to cystic out-pouching, increasing vulnerability for the development of diverticula [9]. The pathogenesis of PTACs is diverse [10]. They may be congenital in origin or acquired later in life [11]. The term “acquired paratracheal cyst” is generally applied to lesions developing in adulthood as a result of focal tracheal wall weakness that may be caused by trauma, infection, high-pressure injuries, long-standing tracheostomy, or obstructive tracheal disease [11]. The differential diagnosis of PTACs includes tracheal diverticula, which are characterized by single or multiple outpouchings of the tracheal wall.

Unlike PTACs, diverticula typically maintain a communication (sometimes very narrow) with the tracheal lumen. Although most PTACs are asymptomatic, rare reports in the literature describe them as tracheal diverticula with symptoms attributable to local mass effect or compression of adjacent structures. Clinical manifestations included chronic cough, non-purulent sputum, recurrent respiratory infections, dyspnea, stridor, dysphagia, odynophagia, and pain in the chest, neck, or right clavicular region [12,13]. Hoarseness has also been reported as a primary symptom in cases where PTACs exert external pressure on the airway, despite normal findings on vocal cord examination [14].

A previous study conducted, where it initially misdiagnosed PTAC as an esophageal diverticulum based on ultrasound findings, showed that the ultrasound showed internal hyperechoic foci caused by reverberation artifact of air, and suggested that in order to distinguish PTAC from esophageal diverticulum on ultrasound, a unique finding of esophageal diverticulum on ultrasound is a hypoechoic rim with or without a multilayered pattern. This finding suggests that the digestive tract is the origin of the lesion (mucosa, submucosa, and muscular layers). Esophageal diverticula are usually found on the left side [9]. And that PTACs are usually located on the right side of the trachea [3], other conditions that should be considered in the differential diagnosis include laryngocele, pharyngocele, Zenker diverticulum, apical lung hernia, apical paraseptal blebs/bullae, and pneumomediastinum [2]. Most PTACs remain asymptomatic; however, symptomatic lesions require management. Initial therapy is conservative, including antibiotics, mucolytics, or bronchodilators. In patients with severe or recurrent manifestations such as compressive symptoms (dyspnea, dysphagia, hoarseness), repeated infections, or chronic cough, surgical excision is indicated [9].

## Conclusions

Paratracheal air cysts are most encountered as tracheal diverticula, which typically demonstrate a discrete communication with the tracheal lumen on bronchoscopy. In contrast, the present case revealed entirely normal findings on both bronchoscopy and upper gastrointestinal endoscopy, with no detectable opening into either the airway or the esophagus. This finding strongly supports the diagnosis of an isolated paratracheal air cyst rather than a diverticulum. The absence of luminal communication not only distinguishes this lesion from the more prevalent tracheal or esophageal diverticula but also underscores the diagnostic value of a multimodal approach. Dependence on cross-sectional imaging alone may lead to misinterpretation, whereas endoscopic correlation is essential to accurately define the nature of such isolated cystic paratracheal lesions.
